# Increased translation in adult mouse striatum is sufficient to induce motor dysfunction

**DOI:** 10.1093/braincomms/fcaf250

**Published:** 2025-06-19

**Authors:** Carla Castany-Pladevall, Jordi Creus-Muncunill, Maria Bergé-Gardeñes, Arantxa Golbano, Verónica Brito, Esther Pérez-Navarro

**Affiliations:** Departament de Biomedicina, Facultat de Medicina I Ciències de la Salut, Institut de Neurociències, Universitat de Barcelona, C/Casanova 143 Barcelona, Catalonia 08036, Spain; Institut D’Investigacions Biomèdiques August Pi I Sunyer (IDIBAPS), Barcelona, Catalonia, Spain; Centro de Investigación Biomédica en Red Sobre Enfermedades Neurodegenerativas (CIBERNED), Madrid, Spain; Departament de Biomedicina, Facultat de Medicina I Ciències de la Salut, Institut de Neurociències, Universitat de Barcelona, C/Casanova 143 Barcelona, Catalonia 08036, Spain; Institut D’Investigacions Biomèdiques August Pi I Sunyer (IDIBAPS), Barcelona, Catalonia, Spain; Centro de Investigación Biomédica en Red Sobre Enfermedades Neurodegenerativas (CIBERNED), Madrid, Spain; Departament de Biomedicina, Facultat de Medicina I Ciències de la Salut, Institut de Neurociències, Universitat de Barcelona, C/Casanova 143 Barcelona, Catalonia 08036, Spain; Institut D’Investigacions Biomèdiques August Pi I Sunyer (IDIBAPS), Barcelona, Catalonia, Spain; Centro de Investigación Biomédica en Red Sobre Enfermedades Neurodegenerativas (CIBERNED), Madrid, Spain; Departament de Biomedicina, Facultat de Medicina I Ciències de la Salut, Institut de Neurociències, Universitat de Barcelona, C/Casanova 143 Barcelona, Catalonia 08036, Spain; Institut D’Investigacions Biomèdiques August Pi I Sunyer (IDIBAPS), Barcelona, Catalonia, Spain; Centro de Investigación Biomédica en Red Sobre Enfermedades Neurodegenerativas (CIBERNED), Madrid, Spain; Departament de Biomedicina, Facultat de Medicina I Ciències de la Salut, Institut de Neurociències, Universitat de Barcelona, C/Casanova 143 Barcelona, Catalonia 08036, Spain; Institut D’Investigacions Biomèdiques August Pi I Sunyer (IDIBAPS), Barcelona, Catalonia, Spain; Centro de Investigación Biomédica en Red Sobre Enfermedades Neurodegenerativas (CIBERNED), Madrid, Spain; Departament de Biomedicina, Facultat de Medicina I Ciències de la Salut, Institut de Neurociències, Universitat de Barcelona, C/Casanova 143 Barcelona, Catalonia 08036, Spain; Institut D’Investigacions Biomèdiques August Pi I Sunyer (IDIBAPS), Barcelona, Catalonia, Spain; Centro de Investigación Biomédica en Red Sobre Enfermedades Neurodegenerativas (CIBERNED), Madrid, Spain

**Keywords:** phospho-4E-BP1, puromycin

## Abstract

Protein synthesis is a process finely regulated in all cell types but specially in neurons as they need rapid changes in protein concentration for synaptic plasticity. Alterations in translation rates have been shown in diseases affecting the brain. In Huntington’s disease (HD), an autosomal dominant neurodegenerative disorder characterized by the presence of motor, cognitive and psychiatric symptoms, we have shown that translation is increased in the striatum contributing to motor symptoms. However, very little is known about how translation modulates motor function in physiological conditions. To study this, we overexpressed a constitutively active mutant form of 4E-BP1 (4E-BP1^F113A^), a translation repressor, in the striatum of wild-type mice and performed motor tests. One month after striatal injection of adeno-associated viral vectors expressing 4E-BP1^F113A^, mice exhibited motor symptoms similar to those observed in the R6/1 HD mouse model. Unexpectedly, *de novo* protein synthesis and 4E-BP1 phosphorylation were enhanced in the striatum of wild-type mice overexpressing 4E-BP1^F113A^. Moreover, the striatum of these animals showed alterations in protein levels of neuronal markers similar to that observed in HD striatum. Altogether, our results indicate that enhanced protein synthesis in the striatum induces neuronal dysfunction and motor symptoms, and reinforce the idea that increased translation is involved in HD pathogenesis.

## Teaser

Castany-Pladevall *et al*. report that the experimental manipulation of protein synthesis, in a particular mouse brain region, the striatum, is enough to worsen motor learning and coordination. The behavioural alterations observed are related to the dysfunction of striatal neurons, which are involved in the regulation of motor function.

## Graphical Abstract

**Graphical Abstract:**
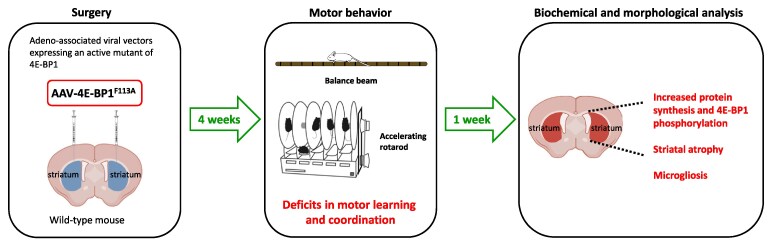

## Introduction

Translation, the proper decoding of mRNAs into proteins, is critical for cellular function, and thus, it requires precise regulation. As a limiting step, initiation, constitutes one of the most highly controlled steps in this process. Initiation of cap-dependent protein synthesis begins with the formation of the eIF4F (eukaryotic initiation factor 4F) or cap-binding complex, which includes the eIF4E protein (5 ‘cap mRNA structure binding protein), the RNA helicase eIF4A and the eIF4G scaffold protein that leads the mRNA to the ribosome. The availability of eIF4E protein is regulated by 4E-BPs (eIF4E-binding proteins), which bind and sequester eIF4E, thus preventing the formation of the eIF4F complex. In turn, the activity of 4E-BPs is finely regulated by phosphorylation, so that when 4E-BP1 is hypo-phosphorylated it binds to eIF4E with high affinity and when hyper-phosphorylated, 4E-BP1 leaves eIF4E free thus allowing cap-dependent translation to occur.^1^

Neurons are especially dependent on rapid changes in the concentration of proteins that are necessary for synaptic plasticity.^2^ An aberrant increase in translation, has been related to psychiatric disorders such as autism and fragile X syndrome.^3-5^ Moreover, increased protein synthesis has been suggested to play a role in neuronal degeneration taking place in Parkinson’s disease associated to LRRK2 mutations.^6^ Similarly, our group has described that increased translation in the striatum is a pathogenic mechanism in Huntington’s disease (HD),^7^ an autosomal dominant neurodegenerative disorder caused by an inherited CAG repeat expansion in the exon 1 of the huntingtin (*HTT*) gene.^8^ In HD, motor dysfunction is the most prominent symptom and is attributed to the degeneration of striatal projection neurons.^9^ However, it is not known whether protein synthesis regulates striatal projection neurons function. To study this, we modulated protein synthesis in wild-type mice striatum by the injection of adeno-associated viral vector (AAV) expressing an active mutant of 4E-BP1.^10,11^

## Materials and methods

### Cultures

Striatal primary cultures were obtained from 18-days-old wild-type mice embryos as previously described.^12^ Cells were seeded onto 6-well plates pre-coated with 0.1 mg/mL poly-D-lysine (P0899, Sigma) at a density of 400 000 cells/cm^2^. Neurobasal medium supplemented with Glutamax (35050–038, Gibco) and B27 (17504-044, Gibco) was used to grow cells in serum-free conditions. Experiments were performed in cultures coming from a minimum of three different litters.

Conditionally immortalized wild-type Htt knock-in striatal cells, ST*Hdh^Q7/Q7^*, were grown as previously described^13^ on 12 mm round glass coverslips and were transfected at 70% of confluence.

### Mice

Male wild-type with C57 background were used for this study. Mice were housed together in numerical birth order with access to food and water *ad libitum* in a colony room kept at 19–22°C and 40–60% humidity, under a 12:12 light/dark cycle. Data were recorded by microchip mouse number. Procedures were carried out in accordance with the National Institutes of Health Guide for the Care and Use of Laboratory Animals, and approved by the local animal care committee of the Universitat de Barcelona (99/01), and Generalitat de Catalunya (99/1094).

### Overexpression of a constitutively active mutant of 4E-BP1 (4E-BP1^F113A^)

Synthesized coding sequence for 4E-BP1 (NM_007918.3) with F113A mutation^10,11^ (Supplementary Fig. 1A) that was originally contained in pCAGGS-4EBP1^F113A^ plasmid^14^ was inserted to pNBM470 vector, containing three human influenza haemagglutinin (HA) epitope tag, generating a fusion protein. HA-4E-BP1^F113A^ plasmid was transfected to ST*Hdh^Q7/Q7^* striatal cells using LipofectamineTM 3000 Transfection Kit (InvitrogenTM; L3000-008).

To over express 4E-BP1^F113A^ in striatal primary neurons and mice striatum we used AAV. The rAAV2/8 plasmids and infectious AAV viral particles containing an empty expression cassette (AAV-null) or with 4E-BP1^F113A^ (AAV-4E-BP1^F113A^) were generated by the *Unitat de Producció de Vectors* from the Centre of Animal Biotechnology and Gene Therapy at the Universitat Autònoma de Barcelona, Catalonia. Striatal primary neuronal cultures were infected at 7 days in vitro (DIV 7) and viral particles were added at a multiplicity of infection 50 000. Striatal primary cultures were infected at 7 DIV and medium was replaced after 24 h with a prewarmed mixture of fresh and the neuronal conditioned medium in a 1:1 ratio.

In 10-week-old wild-type and R6/1 mice, AAV-null or AAV-4E-BP1^F113A^ (2 μL; 1.5 × 10^9^ genomic copies) were bilaterally injected in the striatum at the following coordinates relative to bregma: (i) anteroposterior (AP), +0.8; mediolateral (ML), ±1.8 and (ii) AP, +0.3; ML, ±2, both −2.6 mm ventral from the dural surface.

### Behavioural analysis

General locomotor activity, anxiety and exploratory capacity were evaluated by using the open field test; motor learning was tested with the accelerating rotarod and motor balance and coordination were assessed by using the balance beam test. All procedures were performed as described previously.^7^

### Surface sensing of translation method

Surface sensing of translation method, which is based on the ability of puromycin to label the newly synthesized peptides when administrated at low doses^15^ was applied *in vitro* and *in vivo* and performed as previously described.^16^ Briefly, *in vitro*, 1 µM puromycin was added to confluent cells and incubated at 37°C for 30 min. *In vivo*, corticostriatal coronal slices (400 μm) from R6/1 and wild-type mice were incubated during 1 h in oxygenated artificial cerebrospinal fluid at 32°C, and subsequently treated with puromycin (5 μg/mL) for 45 min. Then, striata were dissected out and flash frozen. Samples were processed for Western blot, as described below and protein synthesis determined by measuring total lane smear signal from 250 to 25 kDa and normalising against α-tubulin or actin.

### Western blot analysis

Proteins obtained from cultures or mice brain samples were processed for Western blot analysis as previously described.^17^ After blocking (Tris buffered saline solution (TBS-T) plus 5% bovine serum albumin and 5% skimmed milk) at room temperature for 1 h, membranes were blotted overnight at 4°C with the corresponding primary antibodies (Supplementary Table 1). After incubation, membranes were washed twice with TBS-T and incubated for 1 h at room temperature with the appropriated horseradish peroxidase conjugated secondary antibody (Supplementary Table 1), and the reaction was finally visualized with the Western Blotting Luminol Reagent (Santa Cruz Biotechnology; Santa Cruz, CA, USA). Western blot replicates were scanned and quantified using a computer-assisted densitometric analysis (Gel-Pro Analyzer, version 4; Media Cybernetics).

### Immunoprecipitation

ST*HdhQ7/Q7* striatal cells transfected with HA-4E-BP1^F113A^ plasmid were collected 48 h after transfection and protein extraction was performed as described above in ice-cold immunoprecipitation (IP) buffer containing 50 mM Tris-HCl (pH 8.0), 1% IGEPAL, 150 mM NaCl and 50 mM NaF. Phosphatase Inhibitor Cocktail Set II (Calbiochem; 524625) and Protease Inhibitor Cocktail (Sigma-Aldrich; 04693116001) were added to avoid protein degradation. Proteins, 300 µg, were incubated O/N at 4°C on a rotating mixer with anti-HA-Tag antibody (Supplementary Table 1; 1:50) or rabbit IgGs (Cell Signalling Technology; 3900) as a negative control. Antibody-antigen complexes were incubated O/N at 4°C with 50 µL of Protein A-Agarose (Santa Cruz Biotechnology; sc-2001) beads and immunoprecipitants were collected by centrifugation (5 min, 4000 rpm at 4°C) and washed three times with IP buffer, IP buffer/PBS (1:1) and PBS, respectively. Proteins were eluted from the beads by boiling the samples for 10 min at 100°C in 4% SDS sample buffer with 10% β-mercaptoethanol. Protein precipitation was then analyzed by Western blot as described above.

### Immunofluorescence

Coronal brain sections (30 μm) were obtained and processed for DARPP-32 immunostaining (1:500; BD Bioscience; 611520; overnight at 4°C) as described elsewhere.^18^ Striatal volume estimation was analyzed by using Cell Profiler Analyst software. Briefly, consecutive sections (an average of 11–14 sections/animal) were visualized on a computer and the perimeter of the striatum was outlined. Striatal volumes were estimated by multiplying the sum of all the sectional areas (μm^2^) by the distance between successive sections (240 μm), as described previously.^19^

### Statistical analysis

As stated in the figure legends, statistical analyses were conducted using the Student's *t*-test for one grouping variable and the two-way ANOVA for multicomponent variables, followed by Bonferroni's *post hoc* test. All results are expressed as the mean and SEM. A 95% confidence interval was used, and values with a *P* < 0.05 were considered as statistically significant.

## Results

### Overexpression of a constitutively active mutant of 4E-BP1 normalizes translation in striatal cells

**Figure 1:**
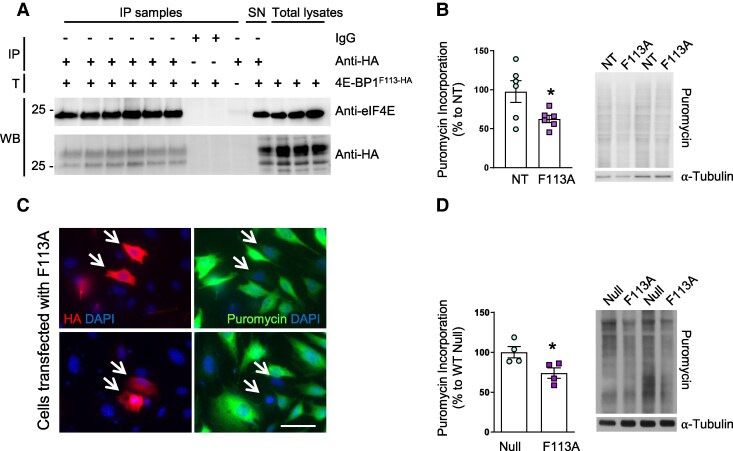
**Overexpressed 4E-BP1^F113A^ decreases puromycin incorporation in STHdh^Q7/Q7^ cells and in striatal primary cultures.** (**A**) Analysis of HA-4E-BP1^F113A^ binding to eIF4E; IP, immunoprecipitation; SN, supernatant; T, Transfection; WB, Western blot. (**B**) Puromycin incorporation, assessed by Western blot, in ST*Hdh^Q7/Q7^* cells 24 h after transfection with HA-4E-BP1^F113A^ (F113A) plasmid, NT: non-transfected cells. (*n* = 6 for all conditions). (**C**) Representative images showing puromycin incorporation in ST*Hdh^Q7/Q7^* cells 24 h after transfection with the HA-4E-BP1^F113A^expressing plasmid. White arrows indicate transfected cells. Scale bar 25 μm. (**D**) Puromycin incorporation in wild-type mice striatal primary neuron cultures 14 days after infection with AAV-Null (Null) or AAV-4E-BP1^F113A^ (F113A) at DIV 7 (*n* = 4 for all conditions). Puromycin incorporation was analyzed by Western blot and Tubulin was used as loading control. Values are expressed as a percentage of (**B**) non-transfected cells or (**C**) AAV-Null treated cultures, and shown as mean ± SEM. Each point corresponds to the value from an individual culture. Representative immunoblots are shown. Two-tailed paired Student’s *t*-test, * *P* < 0.05, compared with cultures (**B**) non-transfected or (**D**) infected with AAV-Null. Corresponding uncropped blots in Supplementary Fig. 4.

In order to analyze whether overexpression of 4E-BP1^F113A^ had the expected effect of increasing binding of 4E-BP1 with eIF4E, we transfected striatal ST*Hdh^Q7/Q7^* cells with a plasmid containing the HA-4E-BP1^F113A^ construct. Binding of HA-4E-BP1^F113A^ to eIF4E was analyzed by IP 24 h after transfection. Indeed, HA-4E-BP1^F113A^ binds to eIF4E (Fig. 1A) and, as expected, puromycin incorporation was reduced in these conditions (Fig. 1B). Moreover, the phosphorylation of 4EB-P1 at Thr37/46 and Ser65 was analyzed by Western blot. We observed that phospho-4E-BP1-HA Thr37/46, but not phospho-4E-BP1-HA Ser65, levels were increased in transfected cells, whereas endogenous levels were not modified (Supplementary Fig. 1B). As ST*Hdh^Q7/Q7^* cells are proliferating cells, we asked whether overexpression of 4E-BP1^F113A^ would exert the same effect in neurons. To that end, mouse striatal primary neurons were infected with AAV-4E-BP1^F113A^ at 7 DIV and puromycin incorporation was analyzed 7 days later. As shown in Fig. 1C, overexpression of AAV-4E-BP1^F113A^ during 7 days decreased puromycin incorporation in striatal primary neurons.

### Overexpression of a constitutively active mutant of 4E-BP1 in wild-type mice striatum induces motor dysfunction

**Figure 2:**
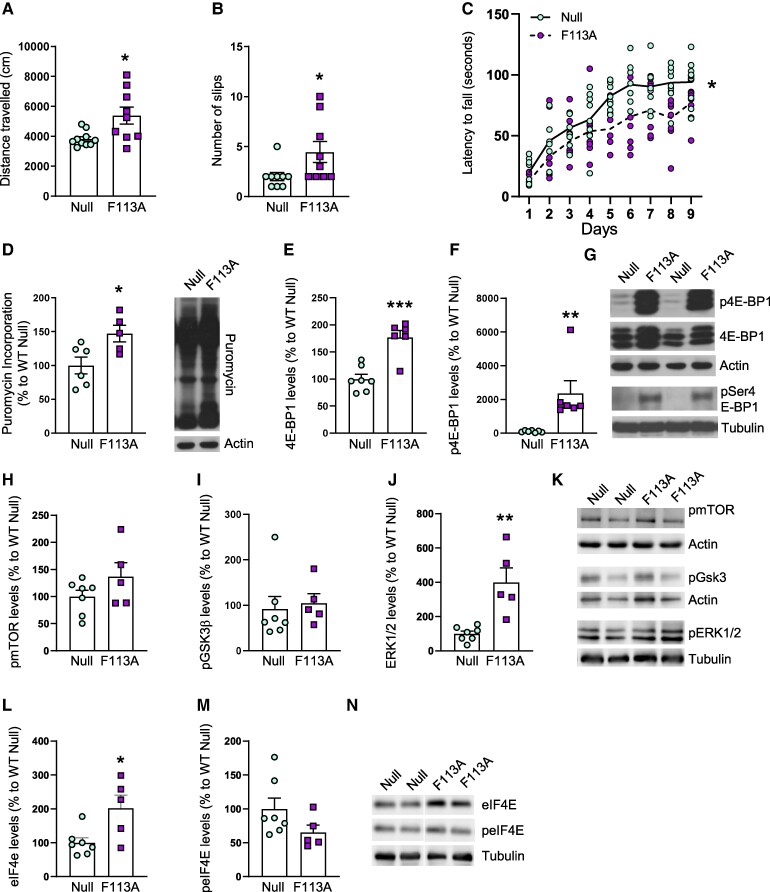
**Intrastriatal injection AAVs expressing 4E-BP1^F113A^ in wild-type mice induces motor dysfunction and alterations in proteins involved in the regulation of translation.** Wild-type mice, at 10 weeks of age, were injected bilaterally with AAV-Null (Null) or AAV-4E-BP1^F113A^ (F113A) in the striatum. Five weeks later, we analyzed motor behaviour, and by Western blot puromycin incorporation and phosphorylation of 4E-BP1, mTOR, GSK3β, ERK and eIF4E. (**A**) Graph showing locomotor activity as the distance travelled (cm) in 10 min in an open field arena (*n* = 9 for all conditions). (**B**) Graph shows the number of slips committed per frame in 2 min in the balance beam test (*n* = 9 for all conditions). (**C**) Accelerating rotarod test was performed for four consecutive days (3 trials per day). Each point corresponds to the value from an individual mouse (*n* = 9 for all conditions). (**D**) Graph shows puromycin incorporation in the striatum of Null (*n* = 6) and F113A (*n* = 5) mice. Results are expressed as a percentage of Null mice. Actin was used as loading control. A representative immunoblot is shown. Graphs showing (**E**) 4E-BP1 and (**F**) p4E-BP1 (Thr37/46) protein levels in the striatum of Null (*n* = 7) and F113A (*n* = 6) mice. (**G**) Representative immunoblots showing pThr37/464 E-BP1, pSer654 E-BP1 and 4E-BP1 levels in the striatum of Null and F113A mice. Actin and tubulin were used as loading control. Graphs show (**H**) pmTOR (Ser 2448), (**I**) pGSK3β (Ser9) and (**J**) pERK (Thr204/Tyr204) protein levels in the striatum of Null (*n* = 7) and F113A (*n* = 5) mice. (K) Representative immunoblots showing pmTOR (Ser 2448), pGSK3β (Ser9) and pERK (Thr204/Tyr204) in all the conditions analyzed. Actin and tubulin were used as loading controls. Graphs show (**L**) eIF4E and (**M**) peIF4E (Ser 209) protein levels in the striatum of Null (*n* = 7) and F113A (*n* = 5) mice. (**N**) Representative immunoblots showing eIF4E and peIF4E (Ser 209) in all the conditions analyzed. Tubulin was used as loading control. In all graphs, values are expressed as a percentage of Null mice. Data are shown as mean ± SEM and each point corresponds to the value from an individual mouse. Data was statistically analyzed by (**C**) two-way ANOVA with repeated measures followed by Bonferroni’s *post hoc* test, *P < 0.05. All other data was statistically analyzed by two-tailed unpaired Student’s *t*-test, *P < 0.05; **P < 0.01; ***P < 0.001; (**G**) mTOR, *P* = 0.1759, *t* = 1.457 and (**I**) GSK3β, *P* = 0.7376, *t* = 0.3445; (**M**) peIF4E, *P* = 0.1337; *t* = 1.632. Corresponding uncropped blots in Supplementary Figs 5–8.

**Figure 3:**
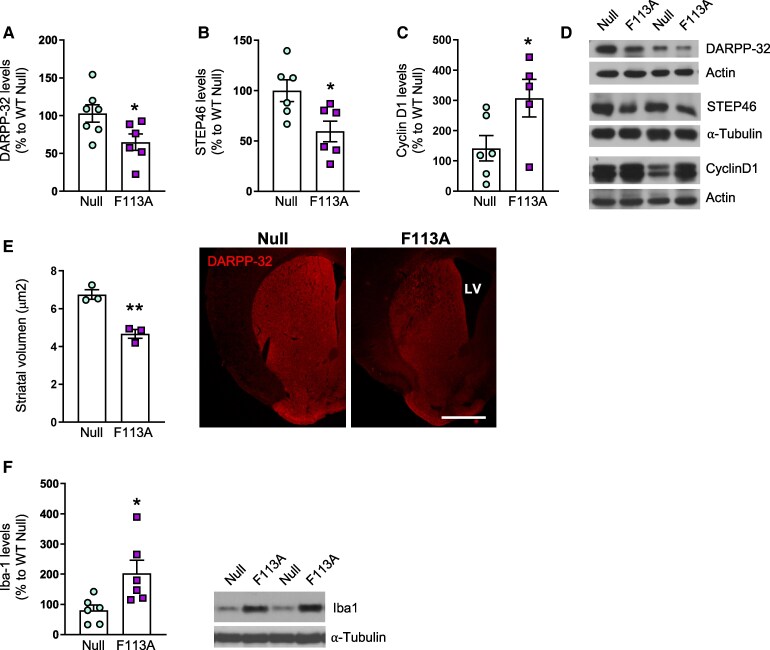
**Intrastriatal injection AAVs expressing 4E-BP1^F113A^ in wild-type mice induces striatal atrophy.** Protein levels of DARPP-32, STEP46, Cyclin D1 and Iba1, and striatal volume were analyzed in the striatum of wild-type mice 5 weeks after intrastriatal injection of AAV-Null (Null) or AAV-4E-BP1^F113A^ (F113A). Graphs showing (**A**) DARPP-32, (**B**) STEP and (**C**) Cyclin D1 levels assessed by Western blot in striatal lysates from Null and F113A mice (DARPP-32, Null *n* = 7 and F113A *n* = 6; Null and F113A, *n* = 6; Cyclin D1, Null *n* = 6 and F113A *n* = 5). Tubulin and actin were used as loading control. Values are expressed as a percentage of Null mice. (**D**) Representative immunoblots showing DARPP32, STEP and Cyclin D1 in all the conditions examined. (**E**) Graph show striatal volume in Null and F113A mice (*n* = 3). Representative images are shown. LV, lateral ventricle. Scale bar 500 μm. (**F**) Graph showing Iba1 levels analyzed by Western blot in the striatum of Null (*n* = 6) and F113A mice (*n* = 6). Tubulin was used as loading control. Values are expressed as a percentage of Null mice. Representative immunoblots are shown. In all bar graphs data are shown as mean ± SEM, and each point corresponds to the value from an individual mouse. Data was statistically analyzed by two-tailed unpaired Student’s *t*-test, *P < 0.05; **P < 0.01. Corresponding uncropped blots in Supplementary Figs 9 and 10.

To specifically modulate protein synthesis in the striatum, 10-week-old wild-type mice were bilaterally injected with AAV-Null or AAV-4E-BP1^F113A^ into the striatum, and motor behaviour was analyzed beginning at 4 weeks after injection (Supplementary Fig. 2A). No differences were observed between wild-type mice injected with AAV-Null or AAV-4E-BP1^F113A^ in terms of body weight or anxiety (Supplementary Fig. 2B). In contrast, distance travelled in the open field, the number of slips in the balance beam and the latency to fall in the accelerating rotarod tests were altered in wild-type mice injected with AAV-4E-BP1^F113A^ (Fig. 2A–C) resembling motor alterations observed in HD mouse models.^7,17^ After performing the behavioural tests, we analyzed whether protein levels of 4E-BP2, the most abundant 4E-BP isoform in the brain,^20^ were altered by the expression of 4E-BP1^F113A^. As shown in Supplementary Figs. 3A, 4E-BP2 protein levels were not different between AAV-Null and AAV-4E-BP1^F113A^ injected striatum. Then, we analyzed puromycin incorporation and 4E-BP1 phosphorylation. Unexpectedly, we detected increased puromycin incorporation in AAV-4E-BP1^F113A^-injected wild-type mice striatum (Fig. 2D) and in line with this result, phosphorylation of 4E-BP1 at Thr37/46 and Ser65 was also increased (Fig. 2E, F). In addition to mTORC1, 4E-BP1 can be phosphorylated by other kinases, such as GSK3β and ERK.^21^ Therefore, we analyzed total and phosphorylated levels of mTOR (Ser2448), GSK3β (Ser9) and ERK 1/2 (Thr204/Tyr 204). In AAV-4E-BP1^F113A^-injected striatum, total levels of mTOR and GSK3β levels where not altered whereas ERK2 levels were decreased (Supplementary Fig. 3B) in comparison to AAV-Null injected striatum. Interestingly, intrastriatal AAV-4E-BP1^F113A^ induced an increase in the levels of phosphorylated ERK1/2 but phospho-mTOR and phospho-GSK3β were not modified (Fig. 2G). Accordingly, protein levels of phospho-p70S6 K (Thr389), which is phosphorylated by mTOR, and also involved in the regulation of translation,^1^ were not altered (Supplementary Fig. 3C) in AAV-4E-BP1^F113A^-injected striatum. Finally, we analyzed total and phosphorylated levels of eIF4E at Ser209 as this phosphorylation modulates translation levels.^22^ We observed that although total levels of eIF4E were significantly increased in AAV-4E-BP1^F113A^-injected striatum, phosphorylated levels were not modified in comparison to AVV-Null injected striatum (Fig. 2H). To analyze if alterations in motor coordination were related to striatal projection neurons degeneration, we analyzed their markers DARPP-32 and STEP46. As shown in Fig. 3A, both DARPP-32 and STEP protein levels were decreased by intrastriatal AAV-4E-BP1^F113A^ injection whereas Cyclin D1, a protein previously shown to increase when 4E-BP is inactivated^23^ was reduced (Fig. 2A). Moreover, the volume of the striatum was reduced in wild-type animals injected with AAV-4E-BP1^F113A^ (Fig. 2B). Finally, we analyzed by Western blot protein levels of Iba1 (ionized calcium binding adaptor molecule 1), as an indicator of microglia activation.^24^ As shown in Fig. 3C, Iba1 levels were highly increased in the AAV-4E-BP1^F113A^ injected striatum in comparison with AAV-Null injected striatum.

## Discussion

Results obtained in the present work support the hypothesis that an aberrant increase in cap-dependent translation in the striatum contributes to neuronal dysfunction leading to motor alterations.

The initial objective of this study was to analyze whether inhibition of translation in the striatum modulates motor coordination. For this purpose, we obtained AAV to selectively overexpress the 4E-BP1^F113A^ construct in mice striatum, a mutant form of 4E-BP1 resistant to mTORC1 phosphorylation, with the purpose of inhibiting cap-dependent translation in this region.^10,11^ First of all, we analyzed the capacity of this construct to inhibit translation *in vitro*. We observed that overexpression of 4E-BP1^F113A^ in striatal ST*Hdh^Q7/Q7^* cells or in striatal primary neurons decreased puromycin incorporation in accordance with previous studies showing inhibition of cap-dependent translation in Neuro2A^14^ and HEK293T cells^25^ overexpressing 4E-BP1^F113A^.

When the constitutively active form of 4E-BP1 was overexpressed in wild-type mice striatum, we unexpectedly observed increased puromycin incorporation instead of a decrease. In line with these observed results, phosphorylation of 4E-BP1 at Thr37/46 and Ser65 was increased. Previous studies show that phosphorylation of 4E-BP1 occurs sequentially, with Thr37 and Thr46 as a priming step followed by phosphorylation of Thr70 and, finally, of Ser65.^26^ This second phosphorylation step results in release from eIF4E and stimulation of translation.^26^ Our findings showing increased levels of 4E-BP1 phosphorylated at Ser65 in wild-type mice striatum overexpressing 4E-BP1^F113A^ suggest that eIF4E is free to form the initiation complex. This can be explained by the fact that the Phe 113 mutation to Ala does not completely abolish insulin-induced phosphorylation of Thr37/46, Ser65 and Thr70,^10^ and 4E-BP1, in addition to mTORC1, can be phosphorylated by other kinases.^21^ Therefore, increased phospho-4E-BP1 in wild-type mice striatum overexpressing 4E-BP1^F113A^ can be the result of the activation of kinases shown to phosphorylate 4E-BP, such as GSK3β^27^ or ERK.^28^ In line with this, we detected increased levels of phosphorylated ERK1/2 in the striatum of wild-type mice overexpressing 4E-BP1^F113A^. Moreover, in the striatum of these animals, we also detected increased levels of eIF4E but not of its phosphorylated form. It is not clear if phosphorylation of eIF4E occurs during or after the eIF4F complex assembly, and depending on the experimental conditions, eIF4E phosphorylation correlates with increased or decreased protein synthesis.^22^

Contrasting results obtained *in vitro* and *in vivo* after overexpression of 4E-BP1^F113A^ could be related to the longer time periods required for *in vivo* overexpression and behavioural analysis experiments and/or the interaction with other cell types. In the *in vitro* experiments, 7 days were sufficient to overexpress 4E-BP1^F113A^ and observe effects on puromycin incorporation. In contrast, for the *in vivo* study, transgene expression after intrastriatal injection of AVVs takes longer^29^ and we decided to wait until 1 month to analyze the effects on motor coordination. We hypothesize that over-expression of 4E-BP1^F113A^ in wild-type mice striatum at short times, and similar to what we observed in cultured striatal neurons 7 days after infection, could be exerting the expected repressive function and thus, translation could be decreased in neurons overexpressing 4E-BP1^F113^. It is possible that cells can cope with a decrease in protein synthesis during a short time (*in vitro* situation), whereas they cannot at longer times. Since dysregulation of translation can be detrimental for neurons,^30^ neurons overexpressing 4E-BP1^F113^ could become dysfunctional. This could activate by an unknown mechanism, possibly involving ERK activation, a compensatory response leading to an increase in phosphorylated 4E-BP1 levels and protein synthesis in those neurons. Moreover, we detected increased levels of the microglial marker Iba1 in AAV-4E-BP1^F113A^ injected striatum suggesting that microglial cells are activated in response to neuronal dysfunction, which could be contributing to exacerbate neuronal dysfunction/death as previously described^31^ Since we were unable to analyze in which cell type translation was being increased, we could not rule out the possibility that this occurs in neurons, microglial cells, or both. Given that increased protein synthesis specifically in microglia alters synapse density,^32^ we cannot exclude the possibility that striatal neuron degeneration may be mediated, at least in part, by altered microglial cells. So far, overexpression of 4E-BP1^F113A^  *in vivo* has been only performed during embryonic development^14^ or in postnatal brain during 5 days.^25^ However, in these studies, 4E-BP1 phosphorylation or translation levels were not examined and beneficial effects observed were attributed to a reduction in protein synthesis.

Increased translation has been related with neuronal dysfunction occurring in several neurological and neurodegenerative diseases.^6,33-35^ In fact, we have previously shown in the R6/1 mouse model of HD that augmented protein synthesis in striatal DARPP-32-positive neurons was involved in their degeneration leading to motor symptoms.^7^ Notably, these animals exhibited altered corticostriatal long-term depression (LTD), a process involved in motor learning. Interestingly, inhibiting this increased translation in the R6/1 mice led to an amelioration of motor dysfunction and a partial restoration of LTD, suggesting its detrimental role in neuronal function.^7^ This is in line with the present findings showing a general increase in protein synthesis in the striatum of wild-type mice overexpressing 4E-BP1^F113A^ that correlated with degeneration of striatal projection neurons, as shown by decreased levels of DARPP32 and STEP, and shrinkage of the striatal volume. Striatal DARPP-32 projection neurons are crucial for motor coordination and thus, their degeneration could be leading to the worsening in the performance on the accelerating rotarod and the balance beam tests that we observed. It has been widely shown that new protein synthesis is necessary for synaptic plasticity in the hippocampus, specifically for long-term potentiation and LTD.^36^ However, few studies have analyzed whether protein synthesis is involved in motor control. Whächter *et al*.^37^ showed that transient inhibition of protein synthesis by intrastriatal injection of anisomycin impairs the acquisition of a novel motor skill in rats, and there is evidence that knocking down 4E-BP2 in mice, alters motor behaviour.^38^ Thus, inhibited or increased translation is detrimental for circuits involved in motor control. This underscores the importance of considering the temporal dynamics when contemplating the modulation of translation as a therapeutic strategy.

In conclusion, despite the unexpectedness of our results, they provide evidence that enhanced protein synthesis in the striatum compromise the functionality of striatal projection neurons involved in the control of movement and reinforces our previous study showing that increased translation is a putative molecular mechanism underlying HD pathogenesis.

## Supplementary Material

fcaf250_Supplementary_Data

## Data Availability

The data that support the findings of this study are available from the corresponding author, upon reasonable request.
