# Supplementary material for: Increased translation in adult mouse striatum is sufficient to induce motor dysfunction
Source: Brain Commun. 2025 Jun 19;7(4):fcaf250. doi: 10.1093/braincomms/fcaf250 (PMC12225667; doi:10.1093/braincomms/fcaf250)
Supplement: fcaf250_Supplementary_Data [file fcaf250_supplementary_data.pdf]

Supplementary Figure 1

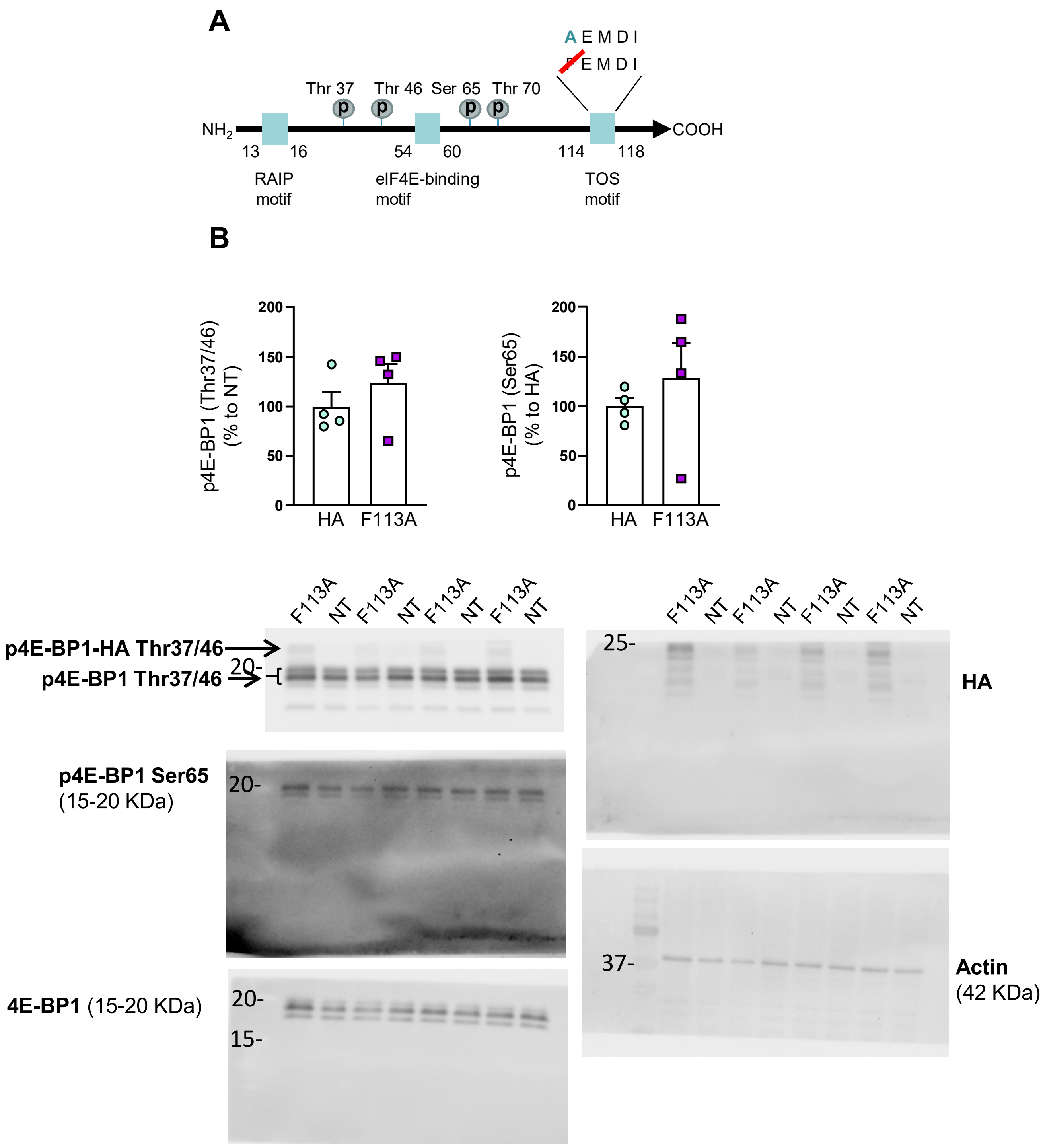

Supplementary Figure 1. (A) Schematic model of 4E-BP1^{F113A} showing phosphorylation sites and the point mutation in the TOS motif. (B) Graphs show endogenous p4E-BP1 Thr37/46 and Ser65 analyzed by Western blot in ST*Hdh*$^{Q7/Q7}$ cells 24 h after transfection with HA-4E-BP1^{F113A} expressing plasmid (F113A). NT: non-transfected cells. Actin was used as loading control. Values are expressed as a percentage of non-transfected cultures and shown as mean ± SEM. Each point corresponds to the value from an individual culture (n = 4). Uncropped blots are shown. Data was statistically analyzed by two-tailed unpaired Student's t-test. Phospho 4E-BP1 Thr37/46, P = 0.3779, t = 0.9516; phospho 4E-BP1 Ser65, P = 0.4679, t = 0.7748. Notice that antobody against 4E-BP1 does not recognize HA-4E-BP1^{F113A}, possibly because it is directed against aa surrounding the point mutation.

Supplementary Table 1 Primary antibodies used for Western blot analysis

Antigen	Host	Dilution	Source / Identifier
4E-BP1	Rabbit	1:1000	Cell Signaling / 9452
4E-BP2	Rabbit	1:1000	Cell Signaling / 2845
4E-BP1 (pThr37/46)	Rabbit	1:1000	Cell Signaling / 9459
4E-BP1 (pSer65)	Rabbit	1:1000	Cell Signaling / 9451
Puromycin (clone 12D10)	Mouse	1:1000	Merck / MABE343
GSK3	Rabbit	1:5000	Cell Signalling / 9315
Cyclin D1	Rabbit	1:1000	Abcam / ab134175
STEP (23E5)	Mouse	1:1000	Santa Cruz Biotech / sc-23892
DARPP-32 (clone 15)	Mouse	1:1000	BD Bioscience / 611520
Iba 1	Rabbit	1:1000	FujiFilm Wako Chemicals (Tokyo, Japan) / 016-2000
GSK3 (pSer9)	Rabbit	1:4000	Cell Signalling / 9336
HA-tag	Rabbit	1:1000	Cell Signaling / 3724
eIF4e	Rabbit	1:1000	Cell Signalling / 9742
eIF4e (pSer209)	Rabbit	1:1000	Cell Signalling / 9741
mTOR	Rabbit	1:1000	Cell Signalling / 2972
mTOR (pSer2448)	Rabbit	1:1000	Cell Signalling / 2971
p70S6K	Rabbit	1:1000	Cell Signalling / 9202
p70S6K (pThr389)	Rabbit	1:3000	Cell Signalling / 9205

ERK	Mouse	1:5000	BD Transduction Laboratories
ERK1/2 (pThr202/Tyr204)	Rabbit	1:1000	Cell Signalling / 9101
α-Tubulin (loading control)	Mouse	1:50000	Sigma Chemical Co. / 6074
Actin (loading control)	Mouse	1:10000	MP Biochemicals / 69100

Supplementary Figure 2

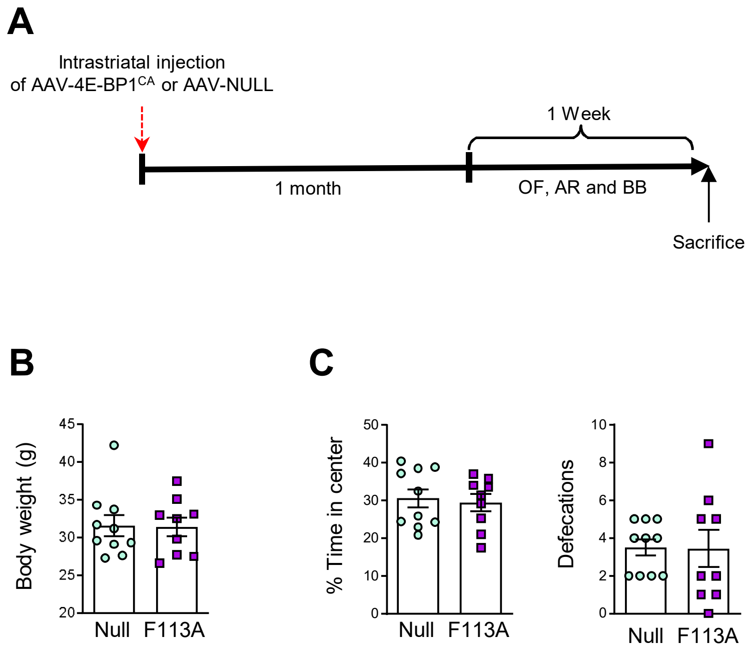

Supplementary Figure 2. Effects of AAV-4E-BP1 F113A intrastriatal injection in wild-type mice. (A) Schematic representation of the experimental design performed. AAV-null or AAV-4E-BP1^{F113A} were injected bilaterally in the striatum of wild-type mice at 10 weeks of age. One month after the injection, behavioral tests were performed during one week and mice were sacrificed at the end of the tests. AR = accelerating rotarod test; BB = balance beam; OF = open field. (B) Body weight at the end of treatment. (C) Percentage of time in the center of the Open field and defecations during the test. All values are shown as mean ± S.E.M and each point corresponds to the value from an individual mouse (Null, n= 10; F113A, n= 9). Data was statistically analyzed by two-tailed unpaired Student's t-test (Body weight: *P* = 0,926, t = 0,09428; Time in the center: *P* = 0,7330, t = 0,3469; Defecations: *P* = 0,9579, t = 0,05353).

Supplementary Figure 3

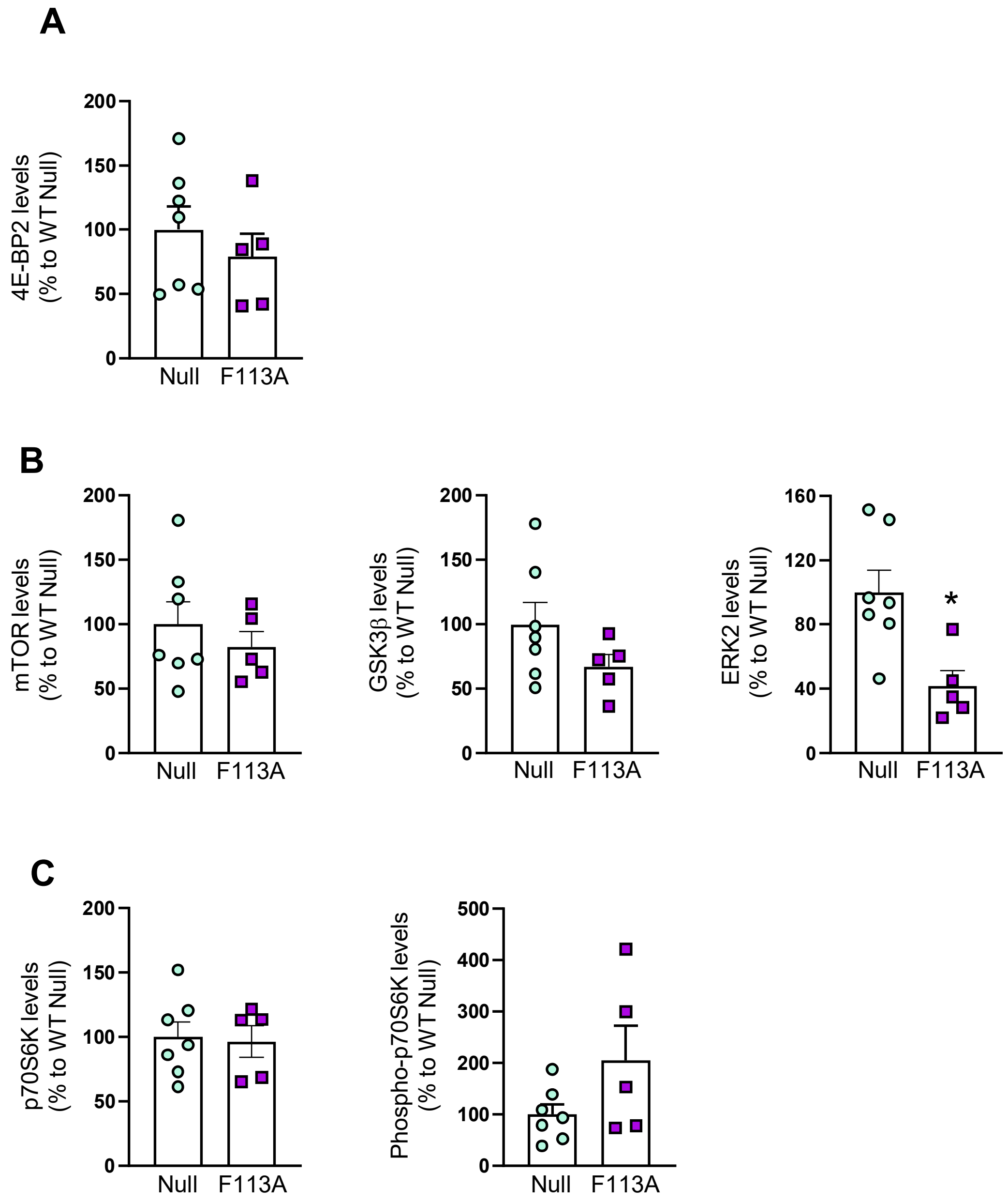

Supplementary Figure 3. Protein levels of several proteins involved in the control of translation after intrastriatal injection AAVs expressing 4E-BP1^{F113A} in wild-type mice. Wild-type (WT) mice, at 10 weeks of age, were injected bilaterally with AAV-Null (Null) or AAV-4E-BP1^{F113A} (F113A) in the striatum. Proteins were analyzed by western blot in striatal lysates 5 weeks after injection. (**A**) 4EBP2, (**B**) mTOR, GSK3β and ERK2 and (**C**) p70S6K and phosphorylated p70S6K (Thr389). Actin or tubulin were used as loading control. See full-size uncropped blots in supplementary figure 6. Values are expressed as a percentage of WT Null mice. In all bar graphs data are shown as mean ± SEM, and each point corresponds to the value from an individual mouse (Null, n = 7; F113A, n = 5). All data was statistically analyzed by two- tailed unpaired Student's t-test, *P < 0.05. (A) P = 0.4396, t = 0.8050; (B) mTOR, P = 0.4638, t = 0.7617; GSK3β, P = 0.1599 t = 1.518. (C) p70S6K P = 0.8395, t = 0.2079; phospho-p70S6K P = 0.1138 t = 1.733.

Supplementary Figure 4

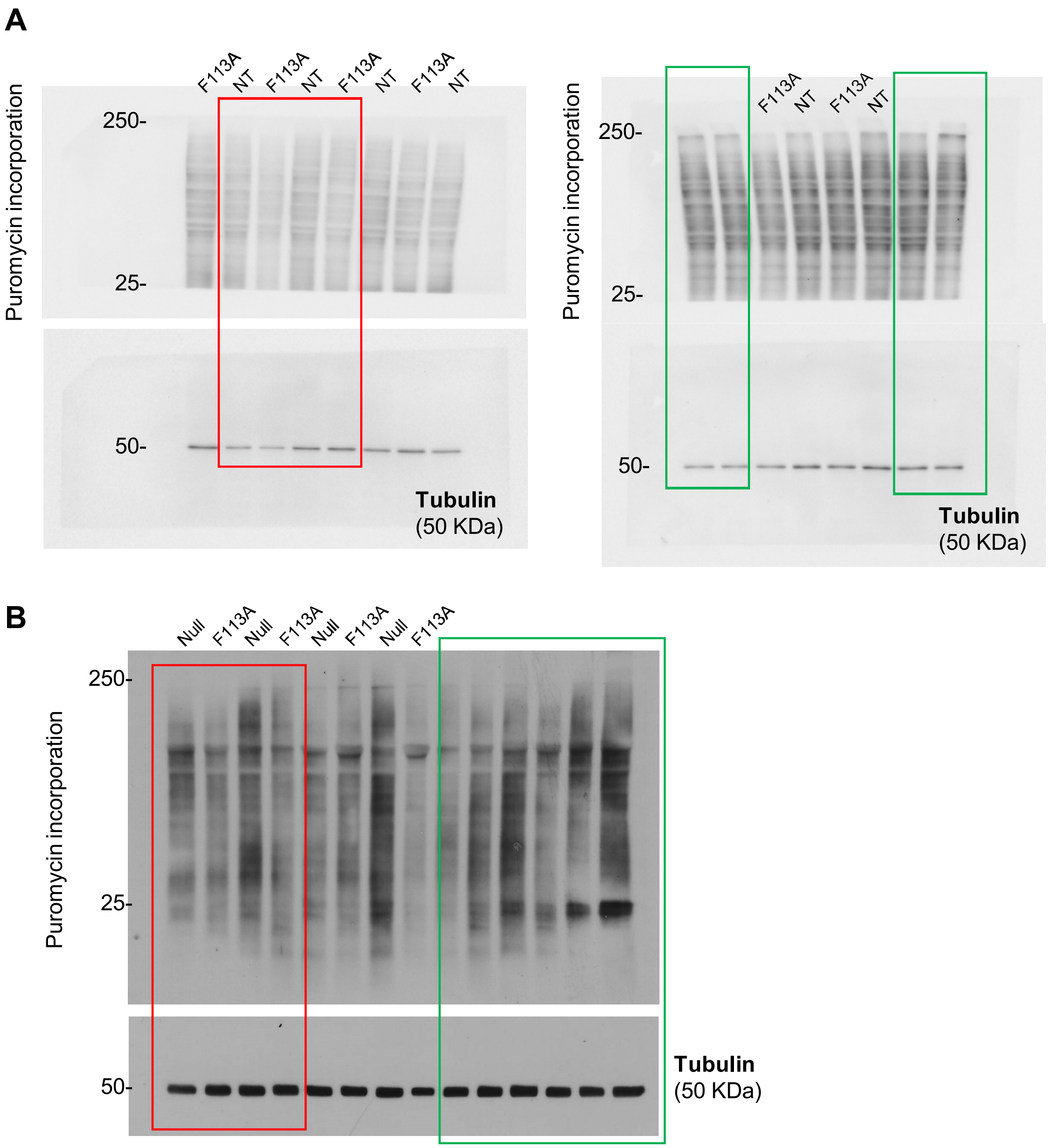

Supplementary Figure 4.- Uncropped blots corresponding to the analysis of (A) puromycin incorporation in (A) ST*Hdh*$^{Q7/Q7}$ transfected with 4E-BP1^{F113A}-HA plasmid (NT: non-transfected; F113A: transfected) and (B) striatal primary neurons infected with AAV-Null (Null) or AAV-4E-BP1^{F113A} (F113A). Tubulin was used as loading control. Rectangles in red indicate representative images shown in Figure 1. Rectangles in green correspond to samples from another study.

Supplementary Figure 5

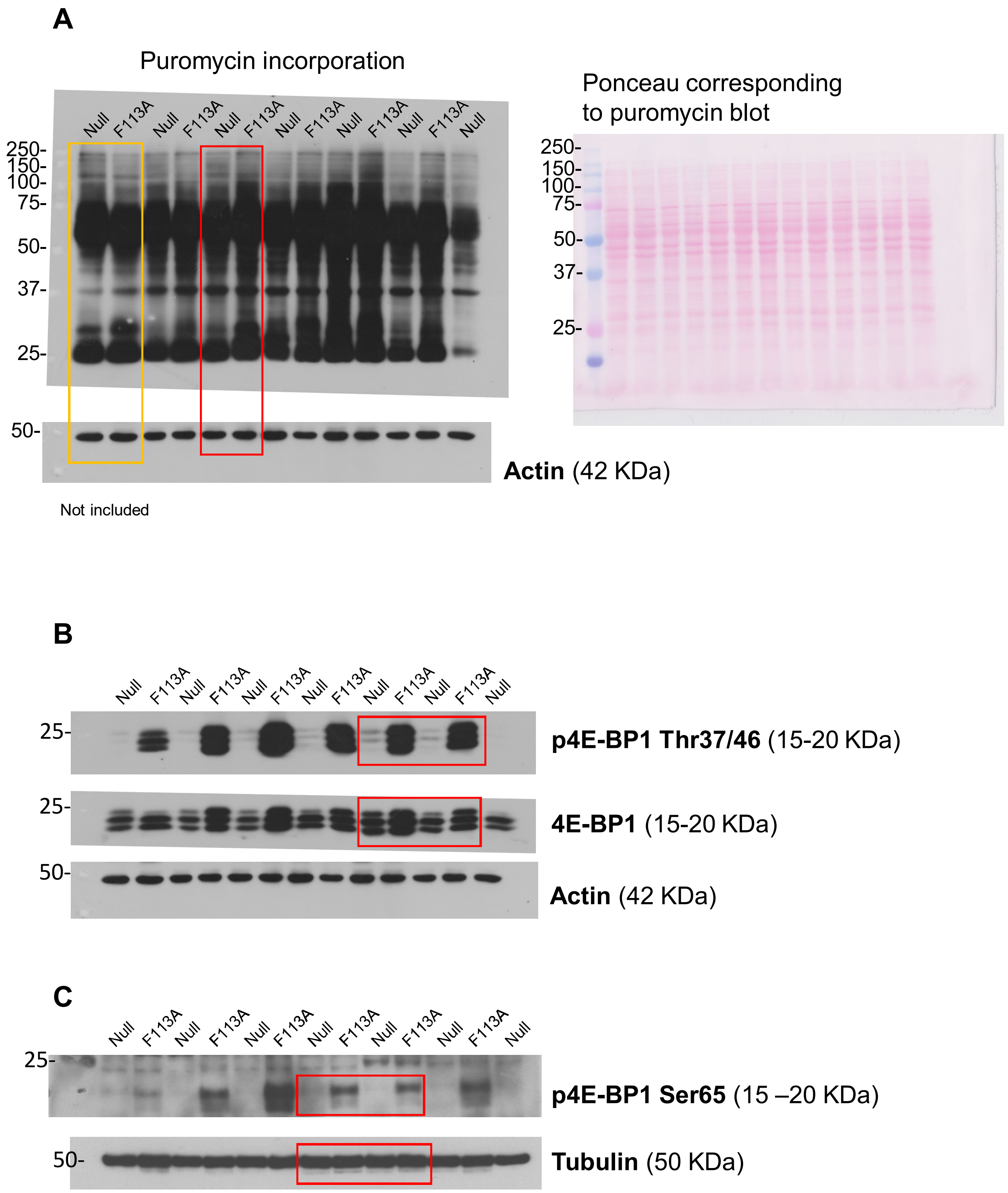

Supplementary Figure 5.- Uncropped blots corresponding to the analysis of (A) puromycin incorporation, (B) phospho Thr37/46 4E-BP1 and 4E-BP1, and (C) phospho Ser65 4E-BP1 in the striatum of wild-type animals injected with AAV-Null (Null) or AVV-4B-BP^{F113A} (F113A). Tubulin or actin were used as loading control. Rectangles in red indicate representative images shown in Figure 2D and G.

Supplementary Figure 6

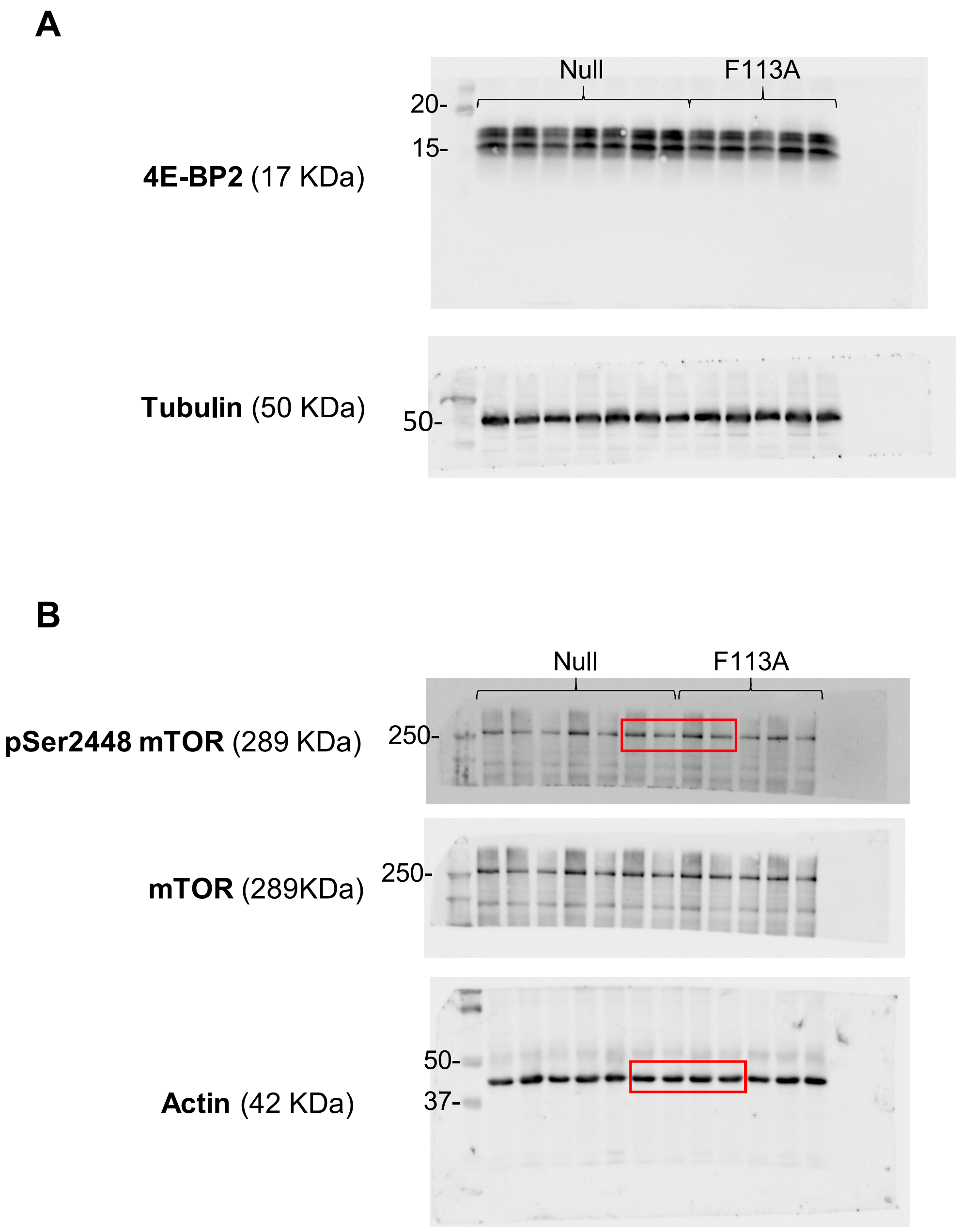

Supplementary Figure 6.- Uncropped blots corresponding to the analysis of (A) 4EBP2 and (B) phospho Ser2448 mTOR and mTOR in the striatum of wild-type animals injected with AAV-Null (Null) or AVV-4E-BP1^{F113A} (F113A). Tubulin or actin were used as loading control. Rectangles in red indicate representative images shown in Figure 2K.

Supplementary Figure 7

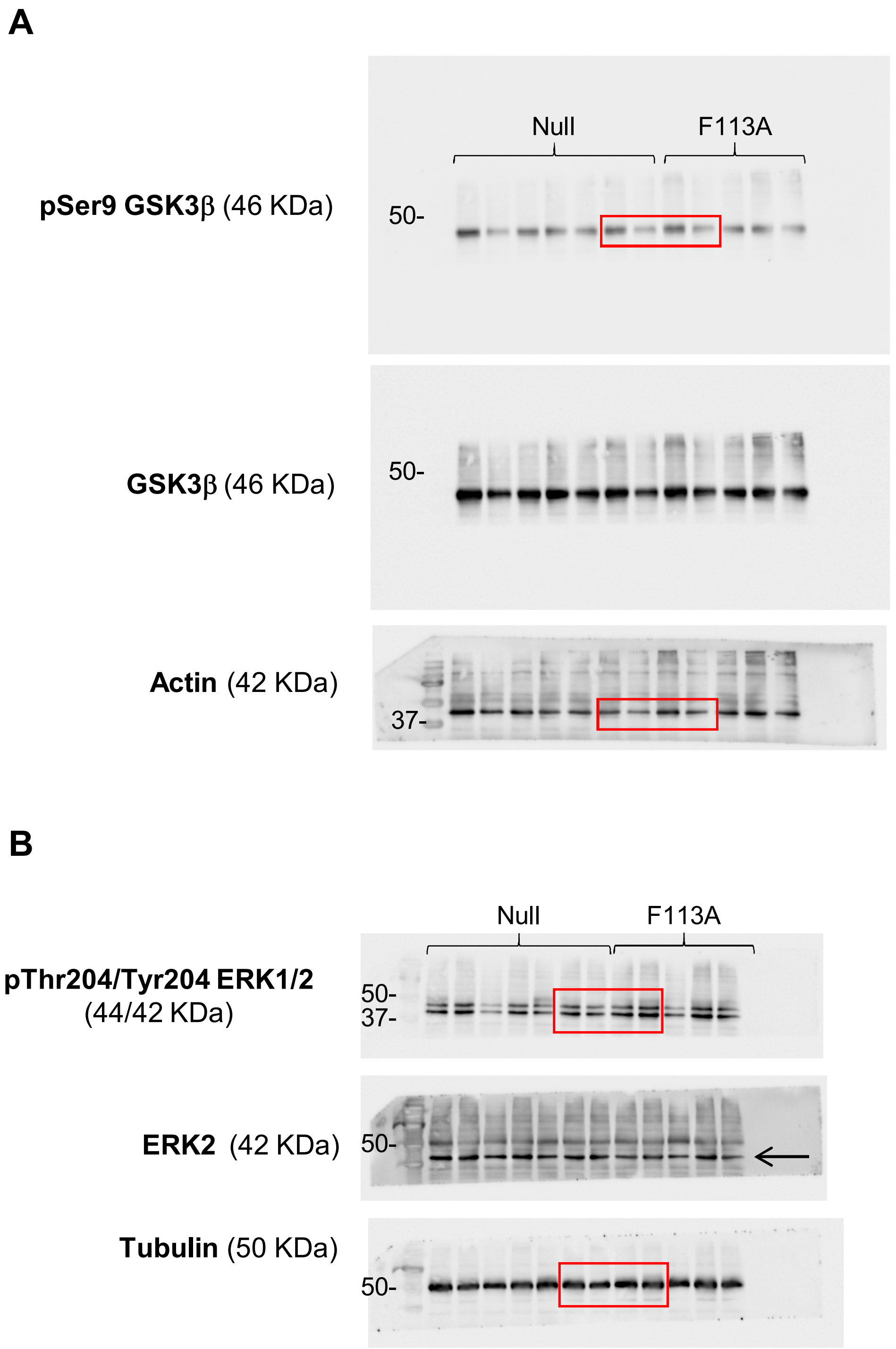

Supplementary Figure 7.- Uncropped blots corresponding to the analysis of (A) Phospho Ser9 GSK3β and GSK3β and (B) phospho Thr204/Tyr204 ERK1/2 and ERK2 in the striatum of wild-type animals injected with AAV-Null (Null) or AVV-4E-BP1^{F113A} (F113A). Tubulin or actin were used as loading control. Rectangles in red indicate representative images shown in Figure 2K.

Supplementary Figure 8

A

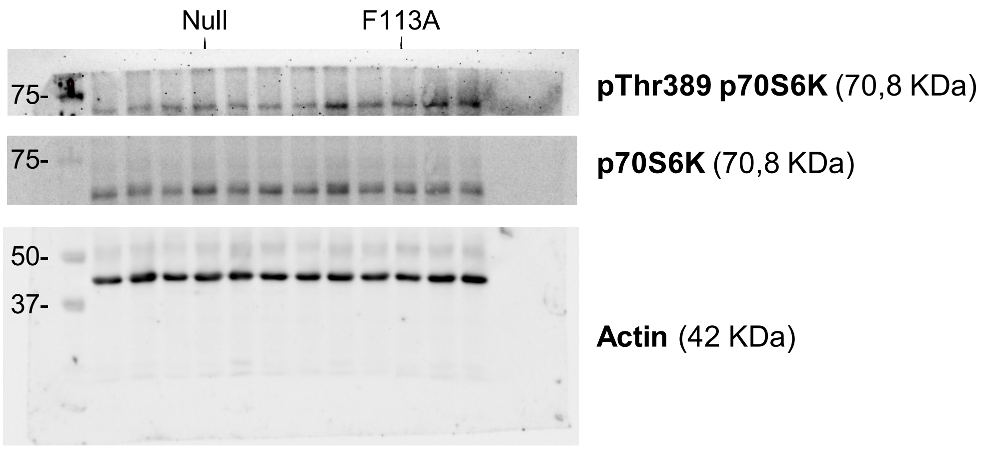

B

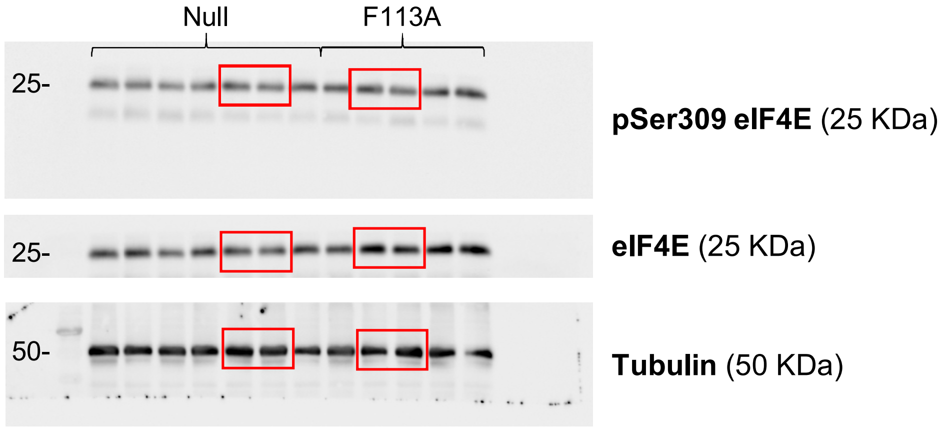

Supplementary Figure 8.- Uncropped blots corresponding to the analysis of (A) phospho Ser389 70S6K and p70S6K, and (B) phospho Ser209 eIF4E and eIF4E in the striatum of wild-type animals injected with AAV-Null (Null) or AVV-4E-BP1^{F113A} (F113A). Tubulin or actin were used as loading control. Rectangles in red indicate representative images shown in Figure 2N.

Supplementary Figure 9

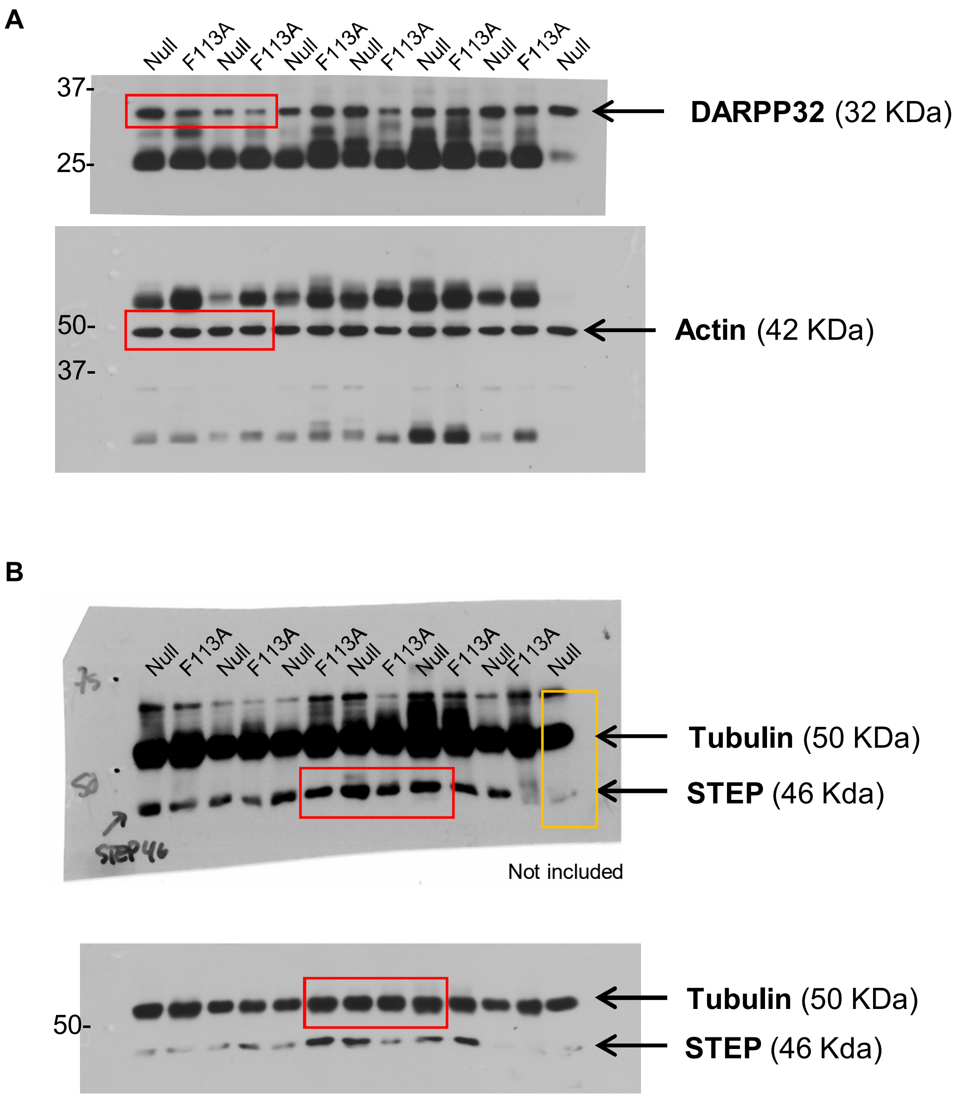

Supplementary Figure 9.- Uncropped blots corresponding to the analysis of (A) DARPP32 and (B) STEP in the striatum of wild-type animals injected with AAV-Null (Null) or AVV-4B-BPF^{113A} (F113A). Tubulin or actin were used as loading control. Rectangles in red indicate representative images shown in Figure 3. Membrane in A was first incubated with anti-puromycin antibody. The other bands correspond to residual bands positive for puromycin.

Supplementary Figure 10

A

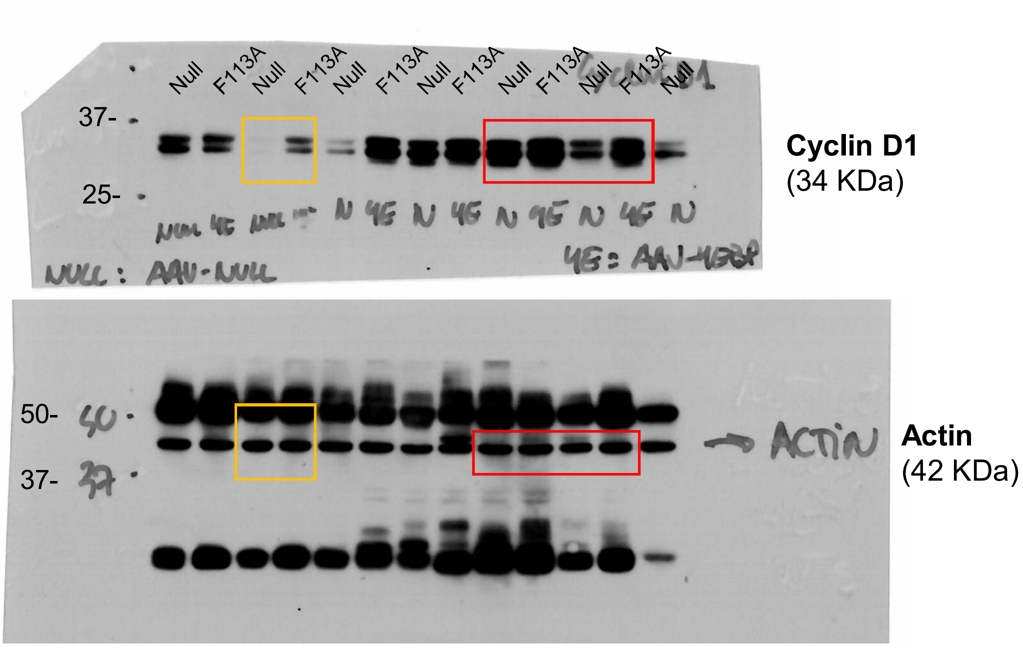

B

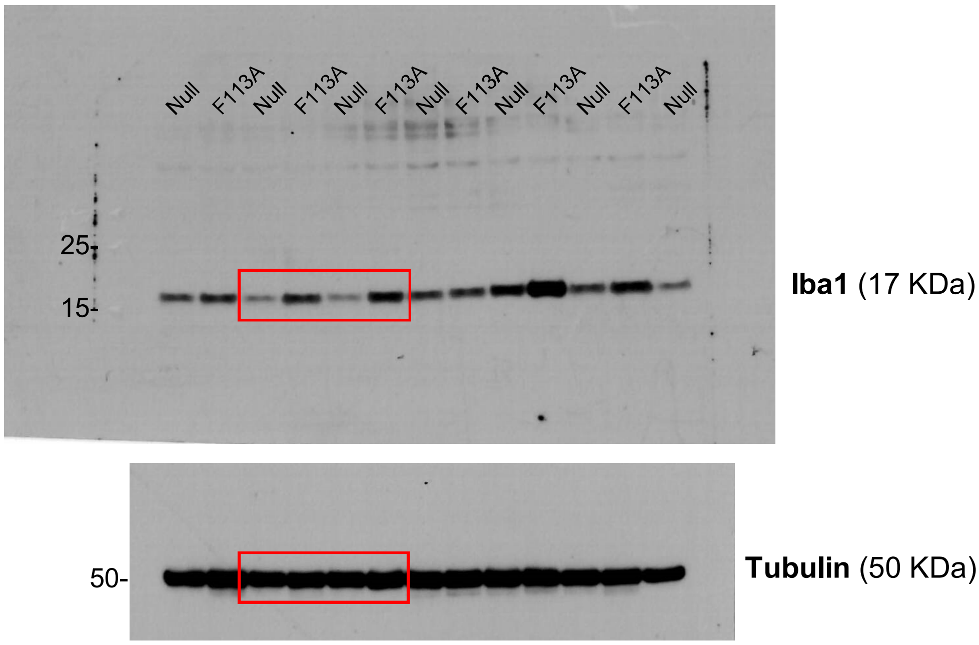

Supplementary Figure 10.- Uncropped blots corresponding to the analysis of (A) Cyclin D1 and (B) Iba 1 in the striatum of wild-type animals injected with AAV-Null (Null) or AVV-4B-BPF^{113A} (F113A). Tubulin or actin were used as loading control. Rectangles in red indicate representative images shown in Figure 3. Rectangles in yellow indicate values not included in the analysis. Membrane in A was first incubated with an antibody against puromycin. The other bands correspond to residual bands from previous incubation.
